# Can integrated safety intervention practices improve sustainable performance? A survey of service organizations

**DOI:** 10.1016/j.heliyon.2024.e31081

**Published:** 2024-05-10

**Authors:** Tinotenda Machingura, Ashleigh Tatenda Muyavu

**Affiliations:** a Lupane State University, P.O. Box 170, Lupane, Zimbabwe; b National University of Science and Technology, P.O. Box AC 939, Ascot, Bulawayo, Zimbabwe

**Keywords:** Safety intervention, Integrated safety intervention, Sustainable performance, Service industry, Developing nation

## Abstract

Despite extensive research on occupational health and safety, the role of safety intervention on performance remains underexplored. Understanding how different integrated safety intervention practices influence sustainable performance could unlock new avenues. This study aimed to investigate the influence of integrated safety intervention practices on economic, social and environmental performance. A survey was conducted in the Zimbabwe service industry and 242 useable responses were obtained. By means of structural equation modelling, we analyzed the effect of management safety intervention, human safety intervention and technical safety intervention on the three dimensions of sustainability. Our findings suggest that safety intervention practices lead to improved sustainable performance. However, the relationship between management safety intervention and sustainable performance is indirect and mediated by human and technical safety intervention. These insights could inform organizations that adopting safety intervention practices is more than compliance with regulations and further shed light on those who are not sure what other benefits besides improving workplace safety can be attained through adopting safety intervention practices.

## 1 Introduction

Safety intervention refers to the adoption of practices that improve safety in workplaces [1,2] which include safety training and administrative procedures [3]. Safety Intervention Practices (SIP) have been widely adopted by organizations seeking to improve their performance and there have been many other reported benefits. However, despite being so important, the impact of SIP on sustainable development has not been fully explored. As a result, most organizations are not aware of how SIP adoption can help them attain enhanced improvements in economic, environmental and social performance (3BL). Although other researchers have explored the impact of SIP on job performance [4] and safety behaviour [3], its influence on sustainability remains unclear. Safety issues are not considered a priority by many firms but a necessity for compliance with regulations and demands by the government [4]. Therefore, by covering the relationship between SIP and 3BL, this study could inform organizations about the benefits of SIP adoption, hence, many organizations might opt to implement it not for safety compliance purposes only but for sustainable gains as well.

Safety intervention has been adopted to improve workplace safety, health of employees, morale of employees, and employee performance [5]. Several studies have been conducted to understand the benefits of adopting SIP. For example, the authors in Ref. [6] reported improved employee participation in the oil industry while the authors in Ref. [7] investigated the role of leadership in improving safety behaviour amongst employees in nuclear power plants and the authors in Ref. [8] assessed the factors that impact safety performance in the construction sector. The reported benefits tend to focus on improving social performance, thus, creating a lot of questions on whether safety intervention has an impact on the other dimensions of sustainability. Methodologies such as Structural Equation Modelling (SEM) can be used to investigate the relationship between SIP and sustainable development by building a model with various safety intervention practices and the 3BL. Two alternatives of SEM exist, partial least squares structural equation modelling (PLS-SEM) and covariance-based structural equation modelling (CB-SEM). Although some studies are based on mathematical models as done in Refs. [9,10], both CB-SEM and PLS-SEM use a statistical approach. CB-SEM requires large and normally distributed data while PLS-SEM do not make any assumptions about the data, hence it is usually preferred [11]. Thus, in this study, the authors address this research gap by using PLS-SEM with environmental, social and economic performance practices as first-order constructs and sustainability as the second-order constructs.

The adoption of safety intervention seems to be widely covered in industries such as manufacturing and construction compared to service industries. A few studies have been done in the service industry, for example, the authors in Ref. [5] investigated the influence of occupational health and safety on employee performance in healthcare services while those in Ref. [4] investigated the impact on job performance in various industries including services. The safety of employees in the service sector is equally important as those in the manufacturing and construction industries as it also affects their well-being and performance. The service sector contributes over 50 % of the Domestic Gross Product (GDP) in most developing countries, for instance, 70 % in China, 51 % in Brazil and 54 % in India [12]. In Zimbabwe, this sector contributes 64 % of the country's GDP, thus, showing how important it is [13]. Therefore, it may be unjust to concentrate on other sectors without paying attention to the biggest GDP contributor. In this research, we focused on understanding how the adoption of SIP by service companies can help them improve not only the safety and health of employees but all the three dimensions of sustainability.

It is vital to understand the importance and performance of each factor towards sustainable performance to determine those with greater importance but lower performance so that they can be improved [14]. For organizations to harness significant improvement in the 3BL, they need to know which factors have a higher contribution to sustainable performance and in case they have limited resources to adopt all practices simultaneously, those with higher importance can be prioritized. Without this understanding, organizations can adopt the SIP randomly and without setting priorities, wasting money on less important practices. Therefore, by assessing the Importance-Performance-Map-Analysis (IPMA), organizations can make informed decisions which enhance their sustainable performance.

SIP can be categorized into Management Safety Intervention (MSI), Human Safety Intervention (HSI) and Technical Safety Intervention (TSI) [3]. It is therefore important to investigate the interrelationships between these practices and their influence towards enhanced sustainable performance. Hence, this study seeks to contribute to the existing knowledge by examining the relationship between managerial, human and technical SIP and the 3BL. MSI being the exogenous construct and sustainable performance as the endogenous construct, it is insightful to explore the relationship between these constructs, whether it is direct, partially mediated or fully mediated by the other constructs which are TSI and HSI. Therefore, in addition to offering assistance to safety managers and organizations looking to enhance their sustainable performance, it is intended that the model in this study will modestly contribute to the fascinating academic discussions that seek to examine these relationships in detail. Henceforth, this research seeks to answer the following questions.

RQ1 Does the adoption of SIP influence the improvement in sustainable performance?

RQ2 Does TSI and HSI mediate the relationship between MSI and sustainable performance?

To address these questions, our study aims to use SEM to assess the inter-relationship between SIP practices and sustainable performance. According to the knowledge of the authors, this is the first study to examine the influence of MSI, HSI and TSI on sustainable performance and further explore their importance and performance. Our findings have far-reaching implications for those organizations that are hesitant to adopt SIP because they are not sure of the full benefits of such adoptions.

## 2 Material and methods

### 2.1 Instrument development

This study utilizes a questionnaire to collect data from different service companies in Zimbabwe. Hence, the development of the questionnaire is very important to make sure that the questions used truly reflect the constructs they are meant to measure. The questionnaire should be well developed and written in simple language so that respondents can answer it without any difficulties and this reduces the missing values [15]. Authors in Ref. [16] indicated that the questionnaire should be as short as possible so that respondents can concentrate thereby improving the reliability of the data. The questionnaire consisted of three sections. Section A focused on the general information about the participants such as their position and years of experience. Section B examined the level of adoption of SIP and the questions were extracted from Refs. [[17], [18], [19]]. The final section assessed the improvement in sustainable performance due to the adoption of SIP. The triple bottom line performance measures were used and the questions for these measures were obtained from Refs. [[20], [21], [22]]. The questions were adopted from the literature to improve the validity [23].

The questions in Section A were open-ended whilst those in sections B and C were closed-ended based on a five-point Likert scale with 1 denoting strongly disagree and 5 indicating strongly agree. The studies from where the research questions were adopted also used the five-point Likert scale and this motivated the authors to adopt the same scale. Before the questionnaire was sent to the respondents, it was pretested by two university professors and three safety and health managers from the service sector with more than 10 years of experience in the study subject. This allowed the authors to modify their questionnaire by removing inappropriate questions, adding omitted questions, and enhancing their logic and clarity [24,25]. After the recommended corrections were made, the final questionnaire was then used to collect data.

### 2.2 Collection of the data

The questionnaire was randomly distributed [6,26] to service companies in Zimbabwe that are registered with the Zimbabwe National Chamber of Commerce (ZNCC). Random sampling was used because, in SEM, high sample sizes are required [27]. Also, it gives participants equal opportunities to be selected [5]. The questionnaire was kept anonymous and could not be traced back to the respondents who completed it, thus reducing bias. 659 questionnaires were distributed using the traditional drop-and-pick method to personnel with higher positions in the safety and health department such as safety managers and supervisors as they have more knowledge of the research area. Emails, WhatsApp messaging and telephone calls were used to communicate with the respondents to know if the questionnaire was completed and ready for collection [23]. 247 responses were initially obtained and after data screening, 5 questionnaires were discarded as they had a lot of missing values. As a result, the valid responses were finally 242 giving a response rate of 36.7 %. Earlier studies have recommended 20 % as the minimum acceptable response rate [28,29] thus, our response rate was good enough. Also, the 10 times rule described in Ref. [30] is used when determining the sample size for SEM. Since the number of structural paths is 8, it means the minimum sample size is 80. Authors in Ref. [8] also argued that the lowest sample size required for SEM is 200. Thus, our sample size of 240 is large enough for analysis.

### 2.3 Non-response bias

The non-response bias was evaluated using the early and late responses approach following the description in Ref. [31]. 10 early and 10 late responses were compared using 5 items of the questionnaire that were chosen at random. The *t*-test results indicated that there was no significant difference at a 5 % significance level meaning that there was no non-response bias, hence further analysis could be done.

### 2.4 Ethical considerations

Each respondent was informed about the study's goal and the authors obtained informed consent from the respondents before they participated in the study. The participation was voluntary and respondents received a guarantee of anonymity. The research was approved by the Lupane State University research ethics committee.

## 3 Literature review

### 3.1 Safety intervention

The goal of safety intervention is to improve safety at workplaces. The adoption of the SIP has many advantages for the organization and workers. These include reduced accident rate, reduced injuries, reduced material damage, reduced equipment damage, increased motivation, decreased absenteeism rate [32], increased safety performance [8], increased employee commitment, increased job satisfaction, increased employee performance [26], foster positive attitudes in employees [33] and increase productivity [26,34,35] which further enhances the profitability. In addition, SIP also reduces the expenses associated with the compensation of employees as a result of workplace accidents and injuries [26]. If organizations fail to adequately support the workers through safety intervention, it could increase worker turnover and reduce worker reliability and loyalty [4].

Safety intervention can be categorized into two levels. MSI is the first level and the second level is comprised of HSI and TSI [3]. The MSI focuses on leadership involvement in safety issues which include administration activities. HSI focuses on the practices that enhance the understanding and knowledge of workers on safety issues that affect them whilst TSI are technical practices that guarantee safety in workplaces [3].

**Fig. 1:**
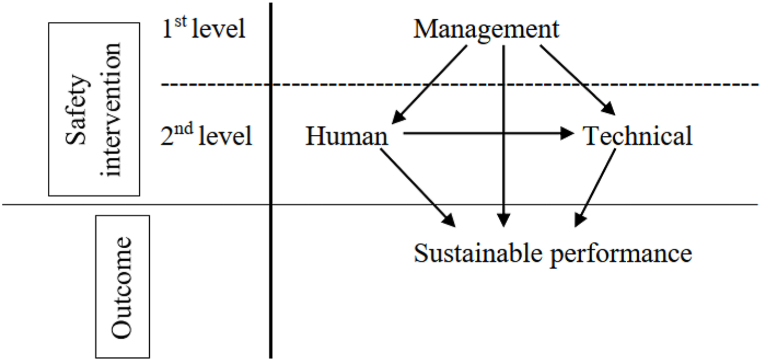
Safety intervention practices.

In this study, we also consider these two levels as shown in Fig. 1. The aim of safety intervention is to have safe workplaces which have an immediate influence on social performance, which can further influence the attainment of environmental and economic performance. Authors in Ref. [36] indicated that safety management differs from one country to another due to the different methods used, unique safety culture and local culture. This makes it difficult for other countries to adopt results obtained in other countries without further research.

**Fig. 2:**
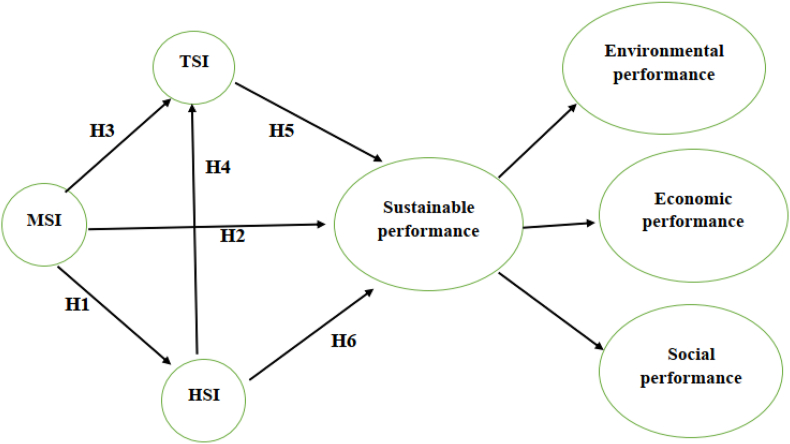
Conceptual model.

This study seeks to understand the interrelationships between MSI, HSI and TSI and their importance in attaining improved sustainable performance. The authors developed a conceptual model shown in Fig. 2 and the proposed hypotheses are discussed in the following sections. One of the variables is sustainable performance which is an endogenous variable while MSI is the exogenous variable, and HSI and TSI are the mediating variables. The models in Refs. [1,3,37] show that MSI influences TSI and HSI and hence these constructs were also used in this study but in this case, measuring their effect on sustainability. The model by Ref. [3] investigated how MSI, TSI and HSI influence the safety behavior in Malaysia's construction industry. In this study, the authors further explored the impact of these variables on the sustainable performance of Zimbabwe's service industry. As the authors in Ref. [23] explained, the results obtained from one industrial sector cannot be adopted by the other sectors without further deliberations. Also, the operating environments differ among countries making it difficult for organizations to generalize the results obtained from other countries. However, such results can be used for benchmarking purposes.

### 3.2 Management safety intervention

The commitment and involvement of management is vital for the successful adoption of safety management and attaining performance improvement [[38], [39], [40]]. Without the support of the leadership, it is difficult for firms to introduce a safety culture and safety practices [25,41]. Thus, management is the foundation of the implementation of safety management programs and safety performance depends on them [6]. According to authors in Ref. [1], management has the capacity to influence the TSI and HSI. MSI and HSI were previously recognized as essential parts of safety and were found to be strongly correlated [42]. Commitment and support by management improve job satisfaction and performance of employees [4], reduce unsafe behaviours [43], and increase the motivation, attitude and dedication of employees [44]. Based on this, the authors formulated the following hypotheses.

H1 MSI has a positive inﬂuence on HSI.

H2 MSI has a positive inﬂuence on TSI.

H3 MSI has a positive inﬂuence on sustainable performance.

### 3.3 Human safety intervention

Employees are responsible for implementing the safety program in organizations; hence their involvement is very important [6]. They need to be trained and equipped with skills for successful safety practices adoption [45]. They also need to be encouraged to embrace the changes brought by safety management programs. Employees can influence the safety performance of organizations by improving workplace health and safety, hence enhancing social performance. When workers neglect or do not pay attention to safety issues at work, human factors become accountable for the majority of industrial accidents [46,47]. Hence, many workplace safety issues are seen to be the result of poor performance by employees [48]. Also, improving the health of employees will reduce their absenteeism at work and increase production hours which then enhance economic performance [4,35]. Authors in Refs. [5,26] added that providing safe working conditions by adopting safety management systems, decreases the rate of employee absenteeism hence directly improving productivity and profitability. Workers are responsible for implementing environmental management practices, and without their full participation and availability, improvements in environmental performance will be jeopardized. Authors in Ref. [1] noted that HSI focuses on improving the knowledge of humans, their competence, behavior, motivation and attitude towards enhanced employee performance. Authors in Ref. [3] noted that HSI practices such as safety training has an effect on TSI practices like safety inspection adoption. Therefore, we can propose the following hypotheses.

H4 HSI has a positive inﬂuence on TSI.

H5 HSI has a positive influence on sustainable performance.

### 3.4 Technical safety intervention

Technical intervention alters the working environment and enhances it to be safer [1]. TSI forms a large part of management planning for reduced accidents and improved employee performance. It includes safer use of equipment and standard operation procedures, thus creating a safer working environment and improving the health of employees, hence, increasing their availability and reducing staff turnover. As explained earlier, such improvements have an impact on sustainable performance. Thus, we proposed the following hypothesis.

H6 TSI has a positive inﬂuence on sustainable performance.

Since TSI and HSI are mediating variables on the relationship between MSI and sustainable development, it is important to investigate the extent of their mediation. Managers should set up strategies that encourage human involvement which will eventually lead to improvement in sustainable performance. Also, by implementing the TSI, organizations are likely to improve their safety and employee performance which further contributes to enhanced sustainable performance. Therefore, we proposed the following hypotheses.

H7 HSI mediates the relationship between MSI and sustainable performance.

H8 TSI mediates the relationship between MSI and sustainable performance.

### 3.5 Sustainable performance

Sustainable performance refers to the competitive advantage in economic earnings that organizations gain by taking into account the influence on the environment and human society [21,49]. Sustainable performance is frequently operationalized using the 3BL which integrates economic, environmental, and social performance, simultaneously [50,51]. The economic dimension is linked to improved productivity and increased ﬁnancial returns while the environmental dimension is concerned with the effect of the firm's activities on the environment and natural systems and the social dimension assesses how organizations' activities impact the workers and societies [21].

Characteristics encountered in workplaces such as excessive workloads, adverse environmental conditions, excessive cold or hot environments, the smell of chemicals, noise, excessive vibration, poor lightning and dust, influence the performance of workers [4]. These conditions lower the concentration of workers when performing their duties leading to a decrease in productivity, poor quality, stress and an increase in cost [52]. If workers know the job procedures and usage of tools it improves their efficiency and effectiveness leading to improved performance [53]. The longer the period the workers spend in an unsafe environment the more it compromises their fitness, hence, affecting performance due to loss of production time [33].

## 4 Results

### 4.1 Proﬁle of the respondents

The details of the respondents who took part in the survey are displayed in Table 1. It shows the companies they belong to, their positions and years of experience in their current position. Most respondents are from the petroleum industry followed by healthcare. The industry with the least number of respondents is information and technology. Also, 33.5 % of the respondents are Safety Health and Environmental (SHE) supervisors, 25.6 % are SHE officers and 20.2 % are SHE Managers. Most of the respondents had a minimum of five years of experience, hence their knowledge was sufficient to comprehensively answer the questions [21]. Table 1 shows the general information about the respondents.

**Table 1:** Demographic characteristics.

Description	Category	Number of respondents	%
Type of industry	Retail	21	8.7
	Hospitality and restaurants	32	13.2
	Petroleum	43	17.8
	Information technology	7	2.9
	Utility	23	9.5
	Refuse	9	3.7
	Maintenance	18	7.4
	Transport and travel	37	15.3
	Cleaning services	13	5.4
	Healthcare services	39	16.1
Position	SHE manager	49	20.2
	SHE officer	62	25.6
	SHE supervisor	81	33.5
	General manager	33	13.6
	CEO	17	7.0
Work experience	0–5 years	14	5.8
	6–10 years	71	29.3
	11–15 years	67	27.7
	16–20 years	58	24.0
	21–25 years	23	9.5
	>25 years	9	3.7

### 4.2 Validity and reliability

Before SEM is performed, it is crucial to check the validity and reliability of the data to assess if it can be used for further analysis. In assessing the internal consistency and reliability, Cronbach's alpha and composite reliability are used. Authors in Ref. [8] emphasized that values above 0.90 show excellent reliability, those between 0.7 and 0.9 indicate high reliability, between 0.5 and 0.7 denote medium reliability and those which are 0.5 and below show low reliability. Table 2 shows that the obtained values ranged from 0.921 to 0.961, hence, the data was deemed to have excellent reliability. Composite reliability values should be above 0.7, likewise, all the values were >0.9, hence acceptable [14]. Convergent validity was explored using Average Variance Extracted (AVE) and values of 50 % or more are suitable [6]. The obtained AVE values were all greater than 0.5, hence, they were accepted. Table 2 shows the reliability and validity results for our study variables, namely MSI, HSI, TSI, environmental, economic, social performance and sustainable performance.

**Table 2:** Reliability and validity results.

	Cronbach's alpha >0.9	Composite reliability >0.7		AVE
		(rho_a)	(rho_c)	
Economic performance	0.921	0.925	0.937	0.68
Environmental performance	0.933	0.959	0.946	0.744
HSI	0.936	0.941	0.949	0.758
MSI	0.934	0.933	0.948	0.754
Social performance	0.961	0.964	0.967	0.786
Sustainable performance	0.936	0.952	0.944	0.575
TSI	0.932	0.941	0.944	0.678

Discriminant validity indicates how a construct is different and unique from other constructs [54]. Cross loadings were used to evaluate discriminant validity where the measures are supposed to have rigorous loadings on their constructs and not on the other constructs which are part of the model [14]. The obtained results, as shown in Table 3, show that all the measurement elements load greatly on their constructs and not on the other constructs, thus satisfying discriminant validity.

**Table 3:** Cross loadings values.

	Economic performance	Environmental performance	HSI	MSI	Social performance	TSI
EC1	**0.776**	0.288	0.264	0.305	0.466	0.394
EC3	**0.78**	0.256	0.278	0.311	0.472	0.4
EC5	**0.824**	0.283	0.32	0.222	0.544	0.407
EC6	**0.798**	0.173	0.43	0.336	0.413	0.354
EC7	**0.84**	0.307	0.482	0.386	0.573	0.327
EC8	**0.901**	0.258	0.362	0.306	0.583	0.422
EC9	**0.848**	0.318	0.363	0.275	0.567	0.3
EN1	0.384	**0.861**	0.063	0.217	0.139	0.018
EN2	0.179	**0.888**	0.033	0.149	0.035	−0.165
EN3	0.154	**0.86**	0.087	0.186	−0.013	−0.162
EN4	0.202	**0.877**	0.133	0.252	0.193	−0.078
EN5	0.223	**0.85**	0.085	0.215	0.175	−0.16
HSI1	0.25	0.108	**0.83**	0.549	0.36	0.488
HSI2	0.439	0.084	**0.871**	0.653	0.51	0.544
HSI3	0.265	0.049	**0.897**	0.672	0.405	0.449
HSI4	0.463	0.112	**0.914**	0.723	0.565	0.589
HSI5	0.413	0.077	**0.84**	0.607	0.513	0.542
HSI6	0.396	0.131	**0.869**	0.619	0.484	0.48
MSI1	0.271	0.222	0.666	**0.892**	0.392	0.505
MSI2	0.336	0.117	0.612	**0.875**	0.433	0.566
MSI3	0.323	0.344	0.638	**0.925**	0.45	0.528
MSI4	0.219	0.19	0.606	**0.91**	0.453	0.592
MSI5	0.42	0.213	0.543	**0.753**	0.499	0.682
MSI6	0.338	0.214	0.746	**0.843**	0.451	0.559
SP1	0.535	0.244	0.507	0.483	**0.855**	0.456
SP2	0.633	0.242	0.502	0.508	**0.918**	0.535
SP3	0.584	0.168	0.472	0.446	**0.907**	0.584
SP4	0.585	0.117	0.506	0.454	**0.94**	0.582
SP5	0.552	0.128	0.455	0.447	**0.929**	0.5
SP6	0.491	0.108	0.443	0.477	**0.832**	0.476
SP7	0.612	0.185	0.585	0.53	**0.903**	0.531
SP8	0.461	0.064	0.416	0.304	**0.795**	0.425
TSI1	0.377	−0.021	0.429	0.476	0.425	**0.818**
TSI11	0.345	−0.102	0.512	0.45	0.357	**0.735**
TSI3	0.29	−0.096	0.304	0.456	0.372	**0.805**
TSI4	0.412	−0.013	0.5	0.601	0.496	**0.893**
TSI5	0.377	−0.088	0.471	0.607	0.535	**0.866**
TSI6	0.311	−0.055	0.41	0.499	0.371	**0.748**
TSI8	0.367	−0.037	0.646	0.632	0.596	**0.881**
TSI9	0.452	0.016	0.57	0.588	0.574	**0.827**

### 4.3 Structural model valuation

The authors started by exploring the collinearity among the constructs using the Variance Inflation Factor (VIF). Authors in Ref. [14] stipulated that the VIF values should be in the range of 0.2–5. The obtained VIF values ranged from 1.793 to 3.450 hence they were satisfactory. The coefficient of determination (R^2^) shows how a dependent construct is explained by the independent constructs that are connected to it. R^2^ values of 26 %, 13 % and 2 % are considered high, medium and low, respectively. Authors in Ref. [30] also added that an R^2^ of 20 % can be considered high depending on the complexity of the model. The R^2^ for many constructs indicates a large effect with only environmental performance showing a medium effect.

The measurement model was further evaluated using the effect size (*f*^2^). The *f*^2^ examines how the R^2^ of the target construct changes when one of the independent constructs is omitted. Values of 0.35 show that the independent variable has a large effect, while 0.15 denotes a medium effect and 0.02 represents a small effect [23]. As shown in Table 4 most relationships showed medium and large effects while that between MSI and sustainable performance showed a low effect.

**Table 4:** *f*^2^ and R^2^ values.

	Economic performance	Environmental performance	HSI	Social performance	Sustainable performance	TSI	R^2^
HSI					0.153	0.151	0.541
MSI			1.178		0.008	0.406	
Sustainable performance	3.277	0.412		4.656			0.367
TSI					0.175		0.465
Economic performance							0.766
Environmental performance							0.175
Social performance							0.823

To evaluate the predictive capabilities of the model, the Cross-Validated Predictive Ability Test (CVPAT) is used which is a remedy to the Stone-Geisser's (*Q*^2^). CVPAT compares the average loss of the model to the average loss of the benchmarks [55]. For the model to have substantive predictive capabilities, the average loss differences between the model and the benchmarks should be significantly below zero [56]. As shown in Table 5, the average loss differences were significantly negative showing high predictive capabilities of the model.

**Table 5:** Predictive capabilities.

	Average loss difference	t value	p-value
Economic performance	−0.167	2.017	0.041
Environmental performance	−0.139	1.981	0.048
HSI	−0.541	3.927	0
Social performance	−0.177	2.838	0.028
Sustainable performance	−0.207	3.632	0
TSI	−0.411	3.174	0.002
Overall	−0.191	3.036	0.003

**Fig. 3:**
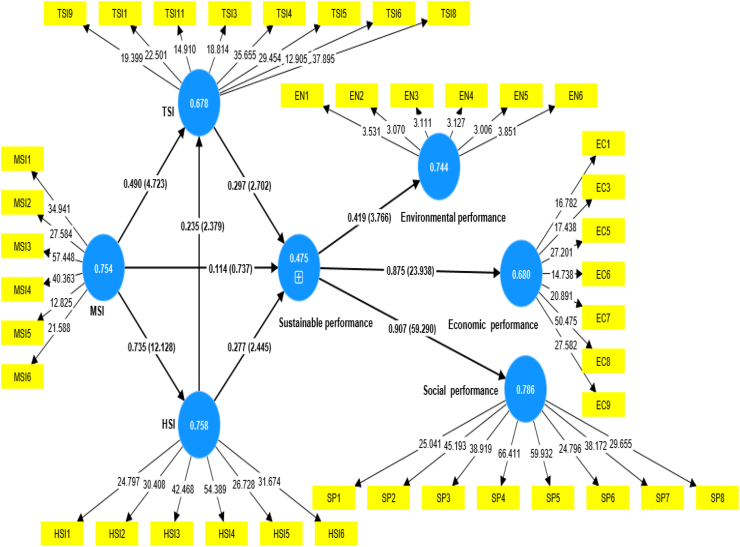
SEM model.

To evaluate whether the hypotheses are valid or not, bootstrapping was performed which gives the t-statistics and p-values. For a hypothesis to be accepted, the t-statistics and p-values should be > 1.96 and < 0.05, respectively. As shown in Table 6, most of the hypotheses met the acceptance criteria, hence, they are supported. Fig. 3 also shows these t-statistics and path coefficients for further clarification.

**Table 6:** Hypotheses decision.

	Path coefficient	t-statistics	p values	Decision
HSI - > Sustainable performance	0.277	2.445	0.015	Accept H4
HSI - > TSI	0.235	2.379	0.017	Accept H5
MSI - > HSI	0.735	12.128	0	Accept H1
MSI - > Sustainable performance	0.114	0.737	0.461	Reject H2
MSI - > TSI	0.49	4.723	0	Accept H3
TSI - > Sustainable performance	0.297	2.702	0.007	Accept H6

### 4.4 Mediation analysis

Using the indirect impacts, we explored the indirect relationship between MSI and sustainable performance to explore the mediation role of TSI and HSI. As discussed earlier, the relationship between MSI and sustainable performance is not supported but as indicated in Table 7, the relationship is indirect, mediated by HSI and TSI, hence the mediation type is indirect only mediation. To further explore the extent of the mediation, the Variance Account Factor (VAF) was employed with values above 20 % showing partial mediation and those greater than 80 % denoting full mediation [57]. The VAF was 66.74 % for HSI as a mediating variable and 36.3 % for TSI, hence, showing partial mediation for both variables. Thus, for firms to fully benefit from safety intervention adoption, there is a need to pay particular attention to both HSI and TSI (see Table 8).

**Table 7:** Mediation analysis.

	Path coefficient	t-statistics	p values	Decision
MSI - > TSI - > Sustainable performance	0.145	2.621	0.009	Accept H8
MSI - > HSI - > Sustainable performance	0.204	2.648	0.008	Accept H7

**Table 8:** Importance performance map analysis.

	Importance	Performance (%)
HSI	0.414	68.28
MSI	0.577	77.71
TSI	0.232	73.74

### 4.5 Importance performance map analysis

IPMA was performed with sustainable development as the target construct. The results show that the importance for MSI, TSI and HSI are 0.577, 0.232 and 0.414, respectively. Thus, the improvement of one of these factors by one unit will improve the sustainable performance by the same margin as the importance of that factor. For instance, an increase in MSI by one unit will improve sustainable performance by 57.7 %. The performance values ranged from 68.28 % to 77.71 % indicating that MSI has a greater performance, than TSI and HSI.

## 5 Discussion

The study investigated the impact of safety intervention on sustainable performance in service industries. Most studies that were done did not pay much attention to sustainable performance, hence, the authors realized the necessity of carrying out this research. Also, most studies focused on the direct impact of TSI and HSI without paying attention to their mediation impact. The authors realized that it was crucial to also examine if an indirect relationship exists which is mediated by TSI and HSI. This study clears doubt on those organizations that are not sure how the implementation of SIP helps them improve their sustainable performance.

The results show that adopting SIP positively influences sustainability. Previous studies have shown mixed results concerning the influence of safety intervention on performance. For instance, the authors in Refs. [4,5,26] noted that safety practices have an impact on performance whilst the authors in Ref. [27] indicated that safety intervention has no impact on productivity performance. In addition, authors in Ref. [8] noted that HSI has no impact on performance whilst MSI significantly contributes to enhanced performance. Through the implementation of safety intervention by the management, the health and safety of workers is improved, the rate of absenteeism is reduced, morale and job performance are enhanced and this eventually leads to improved sustainability.

This study's results also agree with several studies that have noted that occupational safety is positively related to sustainable development. For instance, the authors in Ref. [58] indicated that safety and sustainable development share the same goals and hence they are connected. The authors in Ref. [59] also noted that adopting safety intervention will make workplaces safe, reduce the costs related to accidents, increase the health of workers and increase workplace safety knowledge leading to improved sustainable performance. The authors in Ref. [60] concluded that safety programs and sustainable development are interrelated and with proper implementation, enhanced sustainable performance can be obtained. The relationship between safety culture and sustainable development was explored in Ref. [61], and the authors concluded that safety culture is related to key sustainable performance indicators such as environmental and financial performance. In addition to these studies, we categorized the SIP into MSI, HSI and TSI to fully understand the impact of each factor on sustainable performance and not bunch them into one factor. Also, in this study, the authors investigated the importance and performance of these variables to understand how much each variable contributes towards sustainable performance.

Our results also showed that the relationship between MSI and sustainable development is not supported. Although the authors in Ref. [8] found that the direct relationship is significant, our results agree with the study conducted in Ref. [3] which noted that MSI's goal is to launch the implementation of safety practices in organizations and not necessarily improve performance. Therefore, the relationship between MSI and sustainable performance is indirect through TSI and HSI. To attain improved sustainable performance, it is not sufficient for organizations to focus on MSI only, they should consider TSI and HSI as well. Thus, improvement in sustainable performance is attained by adopting TSI and HSI which are enhanced by MSI implementation. This also agrees with the authors in Ref. [62] who noted that HSI such as safety training mediates the relationship between MSI and safety outcome.

In addition, the authors in Refs. [3,8] concluded that HSI has no impact on safety behaviour and performance. However, in this research, we found that HSI has a significant influence on sustainable development. Humans are responsible for implementing SIP aiming to directly influence social performance. Such improvements have an overall impact on the other dimensions of sustainability. Hence, for organizations to improve their sustainable performance, they need to consider MSI, HSI and TSI as they are all important.

## 6 Conclusion

This study seeks to understand the inter-relationships between MSI, TSI and HSI and examine their direct and indirect impacts on sustainable performance in service industries. Smart PLS 4 was used to analyze the data through SEM. The results show that those organizations that are adopting these practices are able to improve their sustainable performance. Both TSI and HSI were found to have a direct influence on sustainable performance while the impact of MSI is indirect and mediated by TSI and HSI. This sheds more light on those organizations that are not sure how the implementation of SIP can help them improve their sustainable performance. Hence, they may opt to implement these practices not for compliance with regulations only but for sustainable gains as well.

### 6.1 Managerial implications

Since sustainable development is influenced by MSI, TSI and HSI; the allocation of resources should be done appropriately amongst these factors. IPMA shows that MSI is the most important and should be prioritized in case there are limited resources to manage these areas simultaneously. However, organizations should not ignore TSI and HSI as they were found to mediate the relationship between MSI and sustainable performance. The direct relationship between MSI and sustainable performance is insignificant; hence improvement in TSI and HSI will make organizations improve their sustainable performance. More crucially, the study found that the optimal improvement of sustainable performance occurs in a collaborative atmosphere between management and employees.

### 6.2 Limitations and future opportunities of research

The research was conducted within the Zimbabwean context and since the socio-economic conditions and workers' characteristics differ from one country to another, it may be necessary to conduct further research in other countries and results compared with those from this study. Other countries are likely to have safety intervention levels that are different, hence, the results may not apply to these countries. Also, in this study, we focused on the services industries, and we did not include other industries such as manufacturing and construction. Since manufacturing and construction industries are at a higher risk of work-related accidents, it may be insightful to extend this research to these industries. The research also did not separate large organizations from small and medium enterprises. According to authors in Ref. [63], large organizations are at a lower risk of work-related accidents compared to small organizations. It may be beneficial if future studies evaluate if the size of organizations has a moderating effect on this model. This will clearly explore how these organizations can fully benefit from safety intervention.

## Data availability

Data will be made available on request.

## Additional information

No additional information is available for this paper.

## Ethics declaration

All participants provided informed consent to participate in this study.

## CRediT authorship contribution statement

**Tinotenda Machingura:** Software, Resources, Investigation, Conceptualization. **Ashleigh Tatenda Muyavu:** Writing – review & editing, Methodology.

## Declaration of competing interest

The authors declare that they have no known competing financial interests or personal relationships that could have appeared to influence the work reported in this paper.
